# Phenotypes and Outcomes of Heart Failure First Diagnosed During Sepsis: A Single-Center Retrospective Cohort Study Using ICD-10-Based Subtype Classification

**DOI:** 10.3390/jcm15114252

**Published:** 2026-05-31

**Authors:** Arun Gajan Pradeep, Zaineb Zubair, Kaiyu Jia, Mohamad Bahij Moumneh, Mohammad Ennab, Omar Khayat, Ahmed A. Zayed, Martin Miguel I. Amor

**Affiliations:** 1 Department of Medicine, Northwell Health at Staten Island University Hospital, Staten Island, NY 10305, USA; 2 Department of Cardiology, Northwell Health at Staten Island University Hospital, Staten Island, NY 10305, USA

**Keywords:** sepsis, sepsis-induced cardiomyopathy, heart failure with preserved ejection fraction, heart failure with reduced ejection fraction, ICD-10, retrospective cohort

## Abstract

**Background:** Sepsis-associated cardiac dysfunction has historically been characterized as predominantly systolic, yet the burden of heart failure with the preserved ejection fraction (HFpEF) phenotype among patients with heart failure (HF) first documented during sepsis remains poorly defined. **Methods:** In a single-center retrospective cohort of adults (≥18 years) admitted to a tertiary-care hospital between 1 January 2022 and 31 December 2024, we identified patients whose first sepsis diagnosis (ICD-10) coincided with their first HF diagnosis (ICD-10 I50.x) during the same encounter, with no prior HF in the EHR. Patients were classified by ICD-10 subtype as HFpEF, HFrEF, combined, or unspecified; the unspecified group underwent echocardiographic reclassification when LVEF was documented. Demographics, selected comorbidities, peak inflammatory markers, and unadjusted in-hospital and subsequent-encounter outcomes were compared between HFpEF and HFrEF. **Results:** Of 924 patients with HF first documented during sepsis, 438 (47.4%) were ICD-10–coded HFpEF and 405 (43.8%) HFrEF. HFpEF patients were older (74.0 vs. 69.2 years; *p* < 0.001) and more often female (53.0% vs. 37.0%; *p* < 0.001), with higher prevalence of COPD exacerbation (21.5% vs. 13.1%; *p* = 0.004) and hypothyroidism (26.3% vs. 17.5%; *p* = 0.034). HFrEF patients exhibited higher peak lactate (2.88 vs. 2.48 mmol/L; *p* = 0.009) and procalcitonin (8.52 vs. 7.24 ng/mL; *p* = 0.011). Unadjusted in-hospital mortality (28.1% vs. 27.7%; *p* = 0.95), ICU admission (75.8% vs. 74.8%; *p* = 0.80), and length of stay did not differ. Subsequent encounters were descriptively more frequent after HFpEF (11.0% vs. 6.2%). **Conclusions:** ICD-10–coded HFpEF and HFrEF occurred at nearly equal frequency among adults with HF first documented during sepsis, challenging the systolic-centric paradigm. Despite distinct demographic and biochemical profiles, unadjusted in-hospital outcomes did not differ. These descriptive findings, limited by administrative-coding-based phenotype classification, the absence of multivariable adjustment, and unmeasured sepsis-severity and comorbidity variables, are hypothesis-generating and support phenotype-aware prospective study of HF arising during sepsis.

## 1. Introduction

Sepsis is defined as life-threatening organ dysfunction caused by a dysregulated host response to infection and remains a leading cause of in-hospital mortality and long-term morbidity worldwide [1,2]. Cardiac involvement during sepsis is common: between 20% and 60% of critically ill septic patients exhibit some form of myocardial dysfunction during the acute illness, depending on the definition used and the imaging modality applied [3,4,5]. This spectrum of cardiac involvement, often termed sepsis-induced cardiomyopathy (SICM) or sepsis-associated cardiomyopathy, encompasses transient left ventricular (LV) systolic depression, diastolic dysfunction, right ventricular dysfunction, and hyperdynamic states, and is increasingly recognized as a heterogeneous and underrecognized contributor to in-hospital morbidity and post-discharge cardiovascular events [6,7,8].

Historically, the SICM literature has emphasized systolic dysfunction—so-called myocardial “stunning”—mediated by circulating cytokines, mitochondrial dysfunction, calcium-handling derangement, and autonomic dysregulation [6,7,8,9]. More recent imaging-based and translational work has highlighted that diastolic dysfunction is at least as prevalent as systolic dysfunction in this population and may be a stronger predictor of mortality in some cohorts [4,10,11]. In parallel, observational data have demonstrated that pre-existing heart failure (HF), particularly HF with reduced ejection fraction (HFrEF), substantially increases adjusted in-hospital mortality and post-discharge readmission risk in sepsis admissions [12,13]. Despite these advances, comparatively few studies have specifically focused on patients in whom HF is first documented during the sepsis encounter itself, and even fewer have stratified these incident diagnoses by HF phenotype.

Characterizing phenotypes of HF first identified during sepsis is clinically relevant because it may inform hemodynamic management, post-discharge surveillance, and consideration of guideline-directed medical therapy. However, observational studies of HF coded during sepsis admissions face an important interpretive constraint: a first-documented HF diagnosis during sepsis may reflect a truly incident event, transient sepsis-associated myocardial dysfunction, or previously unrecognized chronic HF first recognized during the acute illness. Distinguishing among these possibilities cannot be accomplished using administrative data alone, and any single-snapshot description of phenotype prevalence must be interpreted with that caveat. Within these limits, characterizing the demographic, comorbidity, and biochemical profiles of patients whose HF is first coded during sepsis hospitalization remains a useful starting point for hypothesis generation, particularly given the growing body of evidence framing HFpEF as a systemic inflammatory and microvascular condition [14,15] that may overlap mechanistically with the inflammatory milieu of sepsis.

In this study, we used an EHR-based retrospective cohort drawn from a single tertiary-care center to (1) describe the relative frequency of ICD-10-coded HFpEF, HFrEF, combined, and unspecified HF subtypes among patients whose first HF diagnosis coincided with their first sepsis encounter; (2) compare demographics, selected comorbidities, and peak inflammatory markers between the HFpEF and HFrEF cohorts; and (3) compare unadjusted in-hospital and subsequent-encounter outcomes between the two phenotypes. We framed the analysis as descriptive and hypothesis-generating, and pre-specified that interpretation would be tempered by the limitations of administrative coding, the absence of multivariable adjustment, and the inability to distinguish truly incident HF from previously unrecognized chronic HF unmasked during sepsis.

## 2. Methods

### 2.1. Study Design and Setting

We conducted a single-center retrospective cohort study using EHR data from Staten Island University Hospital (Northwell Health, Staten Island, NY, USA), a tertiary-care academic hospital. The study period spanned hospital admissions occurring between 1 January 2022 and 31 December 2024. The study was reviewed and approved by the Northwell Health Institutional Review Board (Protocol #25-0583) and granted exempt status under 45 CFR 46.104(d)(4)(iii); a waiver of informed consent and a waiver of HIPAA authorization were granted. The manuscript was prepared in accordance with the STROBE statement for reporting of observational studies [16].

### 2.2. Cohort Identification and Eligibility

Adult patients (≥18 years) were identified through a structured query of the EHR for any inpatient encounter during the study period containing an ICD-10 diagnosis code consistent with sepsis or sepsis-spectrum infection, including A40.x (streptococcal sepsis), A41.x (other and unspecified sepsis), R65.20 (severe sepsis without septic shock), and R65.21 (severe sepsis with septic shock). We acknowledge the well-described discordance between ICD-10–coded sepsis and Sepsis-3 clinical criteria [17]; patients identified by these codes were treated as having a clinically adjudicated sepsis diagnosis at the index encounter.

**Figure 1:**
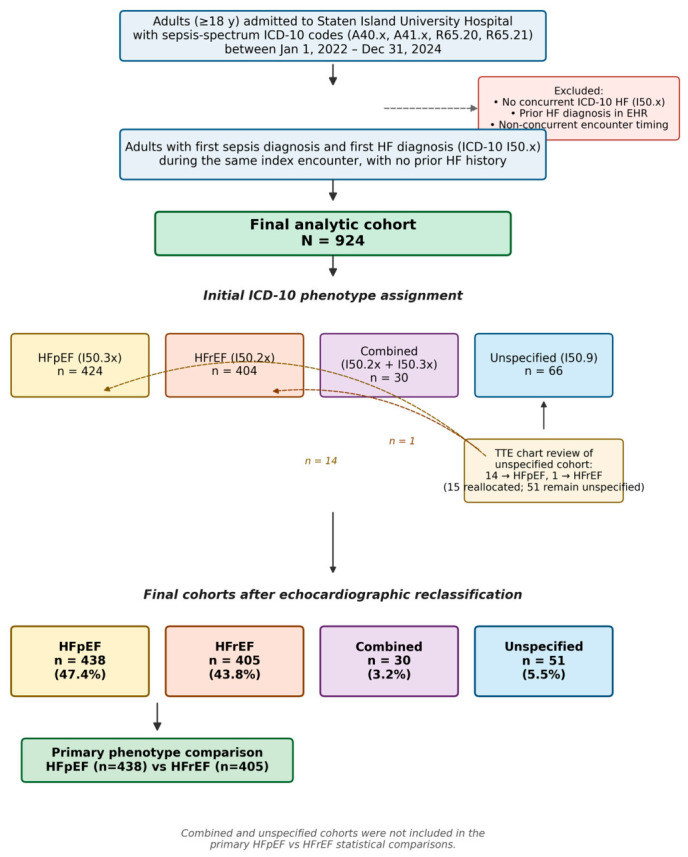
Cohort selection flow diagram. Adults admitted with sepsis-spectrum ICD-10 codes between 1 January 2022 and 31 December 2024 were screened for a concurrent first HF diagnosis. After exclusion of patients without concurrent HF coding, with prior HF history, or with non-concurrent encounter timing, 924 patients formed the analytic cohort. Initial ICD-10 phenotype assignment yielded HFpEF (n = 424), HFrEF (n = 404), combined (n = 30), and unspecified (n = 66). Echocardiographic chart review of the unspecified cohort reallocated 14 patients to HFpEF and 1 to HFrEF based on documented LVEF, yielding final cohorts of HFpEF (n = 438; 47.4%), HFrEF (n = 405; 43.8%), combined (n = 30; 3.2%), and unspecified (n = 51; 5.5%). The primary phenotype comparison was HFpEF versus HFrEF. EHR = electronic health record; HF = heart failure; HFpEF = heart failure with preserved ejection fraction; HFrEF = heart failure with reduced ejection fraction; LVEF = left ventricular ejection fraction; TTE = transthoracic echocardiogram.

Within this sepsis-eligible population, we identified patients with a concurrent ICD-10 diagnosis of HF (I50.x) recorded during the same hospital encounter. To ensure that the HF diagnosis represented an incident event in the available EHR record, we excluded any patient with a prior I50.x diagnosis recorded in any inpatient, outpatient, or emergency department encounter within the Northwell Health EHR predating the index admission. Eligible patients therefore satisfied the following criteria: (1) age ≥ 18 years at index admission; (2) presence of a sepsis-spectrum ICD-10 code at the index encounter; (3) presence of a HF ICD-10 code at the index encounter; and (4) no documented HF diagnosis on any prior encounter in the EHR. To reduce the impact of documentation lag and to confirm encounter-level temporality, charts were manually reviewed to verify that both the sepsis and HF diagnoses corresponded to the index hospitalization. ICD-10 codes were applied at the encounter level rather than to specific calendar days within the admission, and we therefore could not reliably distinguish HF diagnoses present on admission from those first applied later during the same hospitalization; this constraint, and its implications, are addressed in the Section 4.6. The cohort selection process is summarized in Figure 1.

### 2.3. HF Phenotype Classification

Patients were assigned to one of four HF cohorts based on ICD-10 subtype codes recorded during the index encounter: HFpEF (diastolic HF; I50.3x), HFrEF (systolic HF; I50.2x), combined HFpEF/HFrEF (both I50.2x and I50.3x present), or unspecified HF (I50.9 without systolic or diastolic specification). For patients initially coded as unspecified HF, we performed a structured chart review of any transthoracic echocardiogram (TTE) reports obtained during the index admission. When LVEF was documented, patients were reclassified per contemporary American Heart Association/American College of Cardiology/Heart Failure Society of America and European Society of Cardiology definitions [18,19]: LVEF ≤ 40% as HFrEF and LVEF ≥ 50% as HFpEF. On this basis, 15 patients initially coded as unspecified HF were reallocated (14 to HFpEF, 1 to HFrEF), yielding a final unspecified cohort of 51 patients in whom LVEF could not be determined.

We acknowledge that ICD-10–based HF subtype classification is an administrative-data construct that is imperfectly concordant with echocardiographically defined HF phenotype [20,21]. We did not perform a systematic chart-review or echocardiographic validation of HFpEF or HFrEF coding in the primary cohorts, did not extract the proportion of patients undergoing echocardiography during the index admission, and did not record the timing of echocardiographic assessment relative to sepsis onset or whether observed systolic dysfunction persisted beyond the acute phase. Our findings therefore describe patients with ICD-10–coded HF subtypes documented during a sepsis hospitalization, rather than adjudicated clinical HF phenotypes. To partially mitigate misclassification, the unspecified HF cohort underwent the LVEF-based reclassification described above, and patients with combined coding were analyzed as a separate group rather than collapsed into either phenotype.

### 2.4. Variables and Outcomes

Extracted variables included age (years), sex, and selected comorbidities documented during the index encounter, specifically COPD with acute exacerbation, hypothyroidism, and acute kidney injury (AKI). Peak inflammatory and severity markers measured during the index encounter were extracted: C-reactive protein (CRP, mg/L), serum lactate (mmol/L), procalcitonin (ng/mL), and white blood cell count (WBC, ×10^9^/L). The pre-specified variable set did not include additional cardiovascular comorbidities (diabetes mellitus, atrial fibrillation, obesity, chronic kidney disease severity, ischemic heart disease), cardiac biomarkers (NT-proBNP, troponin), detailed echocardiographic indices (E/e′, TAPSE, right ventricular function, valvular disease, pulmonary pressures), or validated severity-of-illness indices (SOFA, APACHE II), vasopressor exposure, mechanical ventilation, or source of infection; the implications of these omissions are addressed in the Limitations Section. When a marker was not measured during the index encounter, the value was treated as missing and the corresponding patient was excluded from comparisons involving that variable on a complete-case basis.

The primary outcome was in-hospital mortality, defined as a discharge disposition of “expired.” Secondary outcomes included intensive care unit (ICU) admission at any point during the index encounter, total hospital length of stay (LOS) measured in hours from admission to discharge or death, and subsequent inpatient or emergency-department encounters within the Northwell Health system following discharge from the index admission. Subsequent encounters were captured for the duration of available follow-up within the study period; no fixed time window (e.g., 30 days, 90 days) was applied, and patients discharged later in the study period therefore had shorter potential follow-up than those discharged earlier. Encounters at non-Northwell facilities could not be captured and may have led to underascertainment of subsequent encounters. The etiology of subsequent encounters (cardiovascular-, infection-, or sepsis-related) was not adjudicated.

### 2.5. Statistical Analysis

Continuous variables were summarized as mean ± standard deviation (SD) when approximately normally distributed and as median with interquartile range otherwise; categorical variables were summarized as counts and percentages. Between-group comparisons of HFpEF versus HFrEF were performed using Student’s *t*-test for normally distributed continuous variables, the Mann–Whitney U test for non-normally distributed continuous variables, and the chi-square test (or Fisher’s exact test where cell counts were small) for categorical variables. The combined and unspecified cohorts were not statistically compared with HFpEF or HFrEF given small sample sizes. The subsequent-encounter analysis was reported descriptively as group proportions; no inferential test was applied given the variable follow-up window and right-censoring at the end of the study period. Two-sided *p* < 0.05 was considered statistically significant. No adjustment was made for multiple comparisons given the descriptive and hypothesis-generating nature of the analysis. Multivariable adjustment for potential confounders (e.g., age, sex, comorbidity burden, severity of illness, organ-support exposures) was not performed and is acknowledged as a primary limitation; all outcome comparisons should therefore be interpreted as unadjusted and exploratory. All analyses were performed in Python 3 using the Pandas and SciPy libraries.

## 3. Results

### 3.1. Cohort Characteristics

During the study period, 924 adult patients met all inclusion criteria for HF first documented during a sepsis hospitalization. After echocardiographic reclassification of the unspecified HF cohort, the final groups were HFpEF (n = 438; 47.4%), HFrEF (n = 405; 43.8%), combined HFpEF/HFrEF (n = 30; 3.2%), and unspecified HF in whom LVEF could not be determined (n = 51; 5.5%). The cohort selection process and final phenotype distribution are summarized in Figure 1. Statistical comparisons in the remainder of this section refer to HFpEF versus HFrEF (Table 1).

Patients with HFpEF were significantly older than those with HFrEF (mean age 74.0 vs. 69.2 years; *p* < 0.001) and were more often female (53.0% vs. 37.0%; *p* < 0.001). The HFrEF cohort was correspondingly predominantly male (63.0%).

### 3.2. Comorbidities

**Figure 2:**
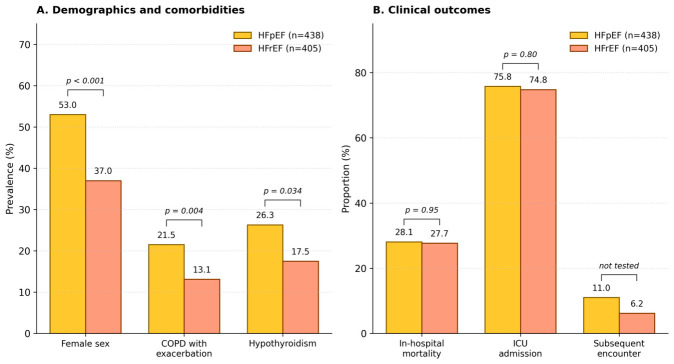
Demographics, comorbidities, and clinical outcomes by HF phenotype. (**A**) Female sex, COPD with acute exacerbation, and hypothyroidism were significantly more prevalent in the HFpEF cohort than in the HFrEF cohort. (**B**) Unadjusted in-hospital mortality and ICU admission did not differ between phenotypes; subsequent encounters within the Northwell Health system during the available follow-up period occurred more frequently after HFpEF, although no inferential test was applied given the variable follow-up window. Outcome comparisons are unadjusted and should be interpreted as descriptive. *p*-values from chi-square tests. COPD = chronic obstructive pulmonary disease; HFpEF = heart failure with preserved ejection fraction; HFrEF = heart failure with reduced ejection fraction; ICU = intensive care unit.

Among the comorbidities captured in our analytic dataset, two differed significantly between cohorts (Figure 2A). COPD with acute exacerbation was more prevalent in the HFpEF cohort than in the HFrEF cohort (21.5% vs. 13.1%; *p* = 0.004), as was hypothyroidism (26.3% vs. 17.5%; *p* = 0.034). AKI occurred frequently in both cohorts and did not differ significantly between groups (*p* = 0.31). Additional cardiovascular comorbidities (diabetes mellitus, atrial fibrillation, obesity, chronic kidney disease severity, and ischemic heart disease) that are clinically relevant to HFpEF–HFrEF differentiation were not captured in the analytic dataset and are discussed as a limitation.

### 3.3. Inflammatory and Severity Markers

Peak serum lactate during the index encounter was higher in HFrEF patients than in HFpEF patients (2.88 vs. 2.48 mmol/L; *p* = 0.009), as was peak procalcitonin (8.52 vs. 7.24 ng/mL; *p* = 0.011). Peak CRP was numerically higher in HFpEF (135.9 vs. 119.6 mg/L) but did not reach statistical significance (*p* = 0.36). WBC distributions overlapped substantially between the two cohorts.

### 3.4. In-Hospital Outcomes

In unadjusted analysis, in-hospital mortality did not differ significantly between cohorts (HFpEF 28.1% vs. HFrEF 27.7%; *p* = 0.95). ICU admission rates were similar (75.8% vs. 74.8%; *p* = 0.80), as was total LOS (449 vs. 459 h; *p* = 0.74) (Figure 2B). Across both phenotypes, in-hospital mortality approached 28%, consistent with the high acute mortality reported in mixed sepsis cohorts complicated by cardiac dysfunction. These unadjusted comparisons should be interpreted as descriptive and do not account for the substantial differences between cohorts in age, sex, comorbidity burden, and unmeasured sepsis severity (see Section 4.6).

### 3.5. Subsequent Encounters

During the available follow-up period within the Northwell Health system, subsequent inpatient or emergency-department encounters occurred more frequently among HFpEF patients than HFrEF patients (11.0% vs. 6.2%; Figure 2B). As described in Methods, no fixed follow-up window was applied, follow-up was right-censored at the end of the study period, and the etiology of subsequent encounters (cardiovascular-, infection-, or sepsis-related) was not adjudicated. This comparison is therefore reported descriptively without an inferential test and should be interpreted strictly as exploratory and hypothesis-generating; the magnitude of the descriptive difference may reflect differential underascertainment or follow-up duration as much as true differences in post-discharge healthcare utilization.

## 4. Discussion

In this single-center retrospective cohort of 924 adults with HF first documented during a sepsis hospitalization, we observed three principal descriptive findings. First, ICD-10–coded HFpEF and HFrEF phenotypes occurred at nearly equal frequency (47.4% vs. 43.8%), challenging a strictly systolic-centric framing of cardiac involvement coded in this clinical setting. Second, the two phenotypes differed substantially in demographic, comorbidity, and acute biochemical profiles: HFpEF patients were older, more often female, and more often had COPD or hypothyroidism, while HFrEF patients had higher peak lactate and procalcitonin. Third, despite these divergent profiles, unadjusted in-hospital mortality, ICU admission, and LOS did not differ between phenotypes; subsequent encounters within the health system, in contrast, occurred descriptively more often after HFpEF. These observations are reported strictly as descriptive and hypothesis-generating, given the constraints of administrative coding, the absence of multivariable adjustment, and the inability to distinguish truly incident HF from previously unrecognized chronic HF unmasked during sepsis.

### 4.1. ICD-10–Coded HF Phenotypes Documented During Sepsis: Context from the SICM Literature

The classical conceptualization of sepsis-induced cardiomyopathy has emphasized cytokine- and mitochondrial-mediated systolic depression with reduced LV ejection fraction [6,8,9]. More recent imaging-based work, however, has consistently identified diastolic dysfunction as at least as common as systolic dysfunction in septic patients, and as a strong correlate of mortality [4,10,11]. The 2025 European Heart Journal state-of-the-art review by Aissaoui and colleagues [8] explicitly characterized SICM as a heterogeneous biventricular syndrome encompassing systolic, diastolic, and right ventricular dysfunction, with no current consensus definition. Our descriptive finding that ICD-10–coded HFpEF and HFrEF were nearly equally represented among HF events first documented during sepsis is conceptually consistent with this broader framing, although our study does not provide direct evidence of sepsis-induced cardiac dysfunction in any individual case. The proportion of our cohort representing transient sepsis-associated myocardial dysfunction, previously unrecognized chronic HF unmasked by acute illness, or truly incident HF cannot be determined from administrative data; this is an important interpretive constraint and is revisited in Section 4.6. Within these limits, the observation that diastolic phenotypes are coded at least as frequently as systolic phenotypes in this clinical setting suggests that any single-phenotype model of cardiac involvement in sepsis is likely to underrecognize a substantial proportion of affected patients.

### 4.2. Phenotypic Divergence: Demographics, Comorbidities, and Inflammatory Signatures

The demographic divergence we observed—older, more female, more comorbid HFpEF versus younger, more male, more biochemically severe HFrEF—mirrors the demographic distinction between HFpEF and HFrEF in non-sepsis populations [14,15,22]. The greater prevalence of COPD with acute exacerbation and hypothyroidism in our HFpEF cohort is consistent with the broader “systemic inflammatory and microvascular” paradigm of HFpEF advanced by Paulus and Tschöpe [14] and elaborated by Schiattarella and colleagues [15], in which chronic comorbidity-driven systemic inflammation, endothelial dysfunction, and microvascular rarefaction promote diastolic stiffness.

Two non-exclusive interpretations of the observed HFpEF predominance warrant consideration. First, sepsis may function as a precipitating systemic inflammatory insult that unmasks or amplifies diastolic dysfunction in a comorbidity-primed substrate, consistent with the systemic–microvascular HFpEF paradigm. Second, and arguably more parsimoniously, the demographic profile of the septic hospitalized population—weighted toward older, multimorbid, and predominantly comorbidity-burdened patients—may itself account for a high background prevalence of HFpEF-typical features independent of any specific sepsis effect. Our descriptive data cannot distinguish between these explanations; both may operate, in differing proportions, across the cohort. Resolution would require prospective cohorts with pre- and post-sepsis echocardiographic and biomarker characterization, which we did not have. The high prevalence of HFpEF coding among our septic patients should therefore be interpreted neither as evidence of a sepsis-specific HFpEF phenotype nor as evidence against it, but as a hypothesis-generating observation that motivates such prospective work.

In contrast, the higher peak lactate and procalcitonin observed in HFrEF align with the classic conception of acute systolic depression as a manifestation of severe circulatory and metabolic stress and high microbial burden [6,23]. An important alternative explanation, however, is that these biochemical differences reflect overall sepsis severity rather than phenotype-specific biology: patients ultimately coded as HFrEF may have been sicker on entry, with higher organ-support exposures and worse early hemodynamic profiles. Because we did not capture validated severity-of-illness indices (e.g., SOFA, APACHE II), vasopressor requirements, mechanical ventilation, or source of infection, we cannot disentangle phenotype-specific from severity-driven differences in inflammatory markers, and the apparent biochemical divergence between phenotypes should be interpreted with that caveat.

### 4.3. Unadjusted Outcomes and Subsequent Encounters: Descriptive Observations

Despite the demographic and biochemical divergence between cohorts, unadjusted in-hospital mortality, ICU admission, and LOS did not differ. We caution explicitly that this represents an unadjusted descriptive comparison: the two phenotypes differ in age, sex, and comorbidity burden, all of which are clinically relevant determinants of outcome in septic populations, and any apparent absence of an outcome difference could mask important phenotype-specific risks once these confounders are accounted for. The absence of a detectable unadjusted difference should not be interpreted as evidence of true prognostic equivalence between phenotypes. Prior administrative-data and prospective cohort work [5,13,24] has demonstrated higher adjusted mortality and post-discharge cardiovascular event risk in survivors of sepsis with HF, and adjusted analyses in prospective cohorts of HF first documented during sepsis are needed to determine whether phenotype-specific outcome differences exist in this population.

The descriptive observation that subsequent encounters within the health system were more frequent after HFpEF (11.0% vs. 6.2%) is interesting and consistent with the long-term “fragility” of the HFpEF phenotype reported in non-sepsis cohorts [25], as well as with broader observations on the cumulative vulnerability of cardiovascular populations to infection-related healthcare exposure [26]. However, our analysis of subsequent encounters has substantial methodologic limitations: we did not apply a fixed follow-up window, follow-up was right-censored at the end of the study period (such that patients discharged later had inherently shorter observation), encounters at non-Northwell facilities could not be captured, and the cardiovascular, infectious, or sepsis-related etiology of subsequent encounters was not adjudicated. This finding should therefore be interpreted strictly as exploratory and hypothesis-generating; it is not evidence of differential post-discharge vulnerability or healthcare utilization patterns. Confirmation would require prospective cohorts with standardized follow-up intervals, time-to-event analysis, and adjudication of encounter etiology.

### 4.4. Clinical Implications

Three tentative implications follow from these descriptive observations. First, given the near-equal frequency of HFpEF and HFrEF coding among patients with HF first documented during sepsis, routine echocardiographic phenotyping during the index sepsis admission—rather than reliance on ICD-10 documentation alone—may improve characterization and downstream management, particularly if combined with cardiac biomarkers and detailed diastolic indices. Second, the demographic and comorbidity divergence between phenotypes may motivate phenotype-specific risk stratification: HFpEF patients warrant particular attention to comorbidity optimization and to follow-up planning, while HFrEF patients warrant attention to early initiation of guideline-directed medical therapy and to the trajectory of recovery of LV systolic function. Third, the higher subsequent-encounter rate descriptively observed in HFpEF, although unadjusted, limited to within-system follow-up, and not adjudicated for etiology, is consistent with broader calls for proactive post-sepsis cardiology surveillance [7] and merits prospective evaluation.

### 4.5. Comparison with Prior Literature

Our work complements and extends prior single-center, multicenter, and administrative-database studies of sepsis and HF. Bou Chebl et al. [27] reported worse outcomes in septic patients with pre-existing HFpEF presenting to a tertiary emergency department but did not focus on HF first documented during the index sepsis admission. Zhu et al. [28] using MIMIC-IV, examined long-term mortality and recurrence in patients with pre-existing HFrEF or HFpEF and sepsis. A 2024 Nationwide Readmission Database analysis [13] demonstrated higher in-hospital mortality and 90-day readmission in HFrEF patients admitted with sepsis. By restricting our cohort to patients with no prior HF history in the available EHR and focusing on ICD-10-coded phenotype-stratified observations within the index encounter, our analysis adds descriptive information to the smaller body of work addressing HF first identified during sepsis, while making explicit the substantial methodologic limits of administrative-coding-based phenotyping in this setting.

### 4.6. Limitations

This study has several important limitations that materially condition the interpretation of our findings. First, the retrospective single-center design limits generalizability, particularly given the demographic specificity of the Staten Island catchment area. Second, sepsis and HF phenotypes were primarily identified using ICD-10 codes. ICD-10 sepsis coding is known to be discordant with Sepsis-3 clinical criteria [17], and ICD-10–based HF subtype classification has imperfect concordance with echocardiographically defined phenotype [20,21]. We did not perform a systematic chart-review or echocardiographic validation of HFpEF or HFrEF coding in the primary cohorts; only the unspecified HF cohort underwent LVEF-based reclassification. The proportion of patients undergoing echocardiography during the index admission, the timing of echocardiographic assessment relative to sepsis onset, and whether observed systolic dysfunction persisted beyond the acute phase were not captured. Our results should therefore be regarded as describing patients with ICD-10–coded HF subtypes rather than adjudicated clinical HF phenotypes, and a sensitivity analysis restricted to patients with echocardiographic confirmation was not feasible with the available analytic dataset.

Third, the term “new-onset” HF, although used in some of the literature, conflates several distinct possibilities in retrospective administrative cohorts: truly incident HF, previously unrecognized chronic HF first identified during the acute illness, and transient sepsis-associated myocardial dysfunction that may or may not persist. Our absence-of-prior-coding inclusion criterion does not reliably distinguish among these scenarios, and a proportion of our cohort—particularly older patients with HFpEF-typical comorbidity profiles—may represent subclinical chronic HF unmasked during sepsis rather than biologically incident HF. We have used the term “HF first documented during sepsis” throughout to make this distinction explicit; resolution would require prospective characterization with pre-sepsis cardiac function data.

Fourth, ICD-10 codes were applied at the encounter level rather than to specific calendar days within the admission, and we could not reliably distinguish HF diagnoses present on admission from those first applied later during the same hospitalization. This temporal indeterminacy precludes mechanistic inference linking HF documentation to a specific phase of the sepsis course.

Fifth, between-group comparisons were unadjusted; we did not perform multivariable regression accounting for age, sex, comorbidity burden, source of infection, vasopressor exposure, mechanical ventilation, or validated severity-of-illness indices, all of which may differ between phenotypes and confound the outcome comparisons. Apparent unadjusted equivalence in mortality, ICU admission, and LOS should not be interpreted as evidence of true prognostic equivalence. The analytic dataset is not currently accessible to the study team for additional modeling; this constraint is acknowledged, and adjusted analyses in subsequent prospective work are a priority.

Sixth, the analytic dataset did not include several clinically important cardiovascular comorbidities relevant to HFpEF–HFrEF differentiation—diabetes mellitus, atrial fibrillation, obesity, chronic kidney disease severity, and ischemic heart disease—and we are unable to retroactively add these variables. Their absence may have biased both phenotype classification (e.g., recognized but uncoded chronic comorbidities predating sepsis) and outcome comparisons. Similarly, validated severity-of-illness indices (SOFA, APACHE II), vasopressor requirements, mechanical ventilation, source of infection, and septic shock stratification beyond ICD coding were not captured; the apparent biochemical divergence between phenotypes (higher lactate and procalcitonin in HFrEF) may therefore reflect differences in overall sepsis severity rather than phenotype-specific biology.

Seventh, we did not capture cardiac biomarkers such as NT-proBNP or troponin, nor detailed echocardiographic indices (E/e′, TAPSE, RV function, valvular disease, pulmonary pressures). These data would have substantially strengthened phenotype characterization, particularly the distinction between acute inflammatory myocardial dysfunction and decompensated underlying cardiac disease, and their absence is a meaningful limitation of the present work.

Eighth, multiple comparisons were performed without formal correction; nominally significant findings should be interpreted accordingly. Peak inflammatory markers were extracted on a complete-case basis without imputation; missingness, particularly for procalcitonin, may have biased estimates. Variable-specific missing-data counts were not separately tabulated.

Finally, subsequent encounters were captured within a single health system without a fixed follow-up window, leading to right-censoring and underascertainment of out-of-system encounters; the etiology of subsequent encounters (cardiovascular, infectious, or sepsis-related) was not adjudicated and a time-to-event analysis was not performed. The 11.0% versus 6.2% comparison is descriptive rather than inferential.

## 5. Conclusions

Among adults with HF first documented during a sepsis hospitalization at a single tertiary-care center, ICD-10-coded HFpEF and HFrEF phenotypes occurred at nearly equal frequency. The two phenotypes differed in demographic profile, comorbidity burden, and peak inflammatory markers, with HFpEF patients older, more often female, and more often comorbid with COPD or hypothyroidism, and HFrEF patients exhibiting higher peak lactate and procalcitonin. In unadjusted descriptive analysis, in-hospital mortality, ICU admission, and LOS did not differ significantly between phenotypes, and subsequent encounters within the health system occurred descriptively more often after HFpEF. These findings are exploratory and hypothesis-generating and are subject to important limitations—including ICD-10-based phenotype classification without systematic echocardiographic validation, the inability to distinguish truly incident HF from previously unrecognized chronic HF unmasked during sepsis, the absence of multivariable adjustment, and the lack of cardiac biomarkers, detailed echocardiographic indices, and validated sepsis-severity measures. They should be interpreted neither as evidence of phenotype-specific mechanisms nor as evidence of prognostic equivalence between phenotypes. They do, however, support a phenotype-aware approach to the characterization of HF arising during sepsis and motivate prospective cohorts incorporating systematic echocardiographic phenotyping, cardiac biomarkers, validated sepsis-severity indices, standardized post-discharge follow-up, and adjusted outcome modeling.

## Figures and Tables

**Table 1:** Demographics, comorbidities, peak inflammatory markers, and outcomes of patients with ICD-10–coded HFpEF versus HFrEF first documented during a sepsis hospitalization (n = 843 with classified phenotype). All comparisons are unadjusted and should be interpreted as descriptive. *p*-values from Student’s *t*-test, Mann–Whitney U test, or chi-square test as appropriate. No inferential test was applied to subsequent encounters given the variable follow-up window. COPD = chronic obstructive pulmonary disease; CRP = C-reactive protein; HFpEF = heart failure with preserved ejection fraction; HFrEF = heart failure with reduced ejection fraction; ICU = intensive care unit. The combined (n = 30) and unspecified (n = 51) cohorts are not displayed.

Characteristic	HFpEF (n = 438)	HFrEF (n = 405)	*p*-Value
Age, mean years	74.0	69.2	<0.001
Female sex, %	53.0	37.0	<0.001
COPD with exacerbation, %	21.5	13.1	0.004
Hypothyroidism, %	26.3	17.5	0.034
Peak CRP, mg/L	135.9	119.6	0.36
Peak lactate, mmol/L	2.48	2.88	0.009
Peak procalcitonin, ng/mL	7.24	8.52	0.011
In-hospital mortality, %	28.1	27.7	0.95
ICU admission, %	75.8	74.8	0.80
Length of stay, hours	449	459	0.74
Subsequent encounter, %	11.0	6.2	—

## Data Availability

The data underlying this study are not publicly available because they contain protected health information governed by HIPAA. De-identified summary data may be made available upon reasonable request to the corresponding author and with the approval of the Northwell Health IRB.
